# Histopathology in HCM

**DOI:** 10.21542/gcsp.2018.20

**Published:** 2018-08-12

**Authors:** Beatriz San Millán Tejado, Cristina Jou

**Affiliations:** 1 Servicio de Anatomía Patológica, Neuropatología, Hospital Álvaro Cunqueiro, Vigo, Spain; 2 Servicio de Anatomía Patológica, Unitat patología Neuromuscular, Hospital Sant Joan de Deu, Barcelona, Spain

## Introduction

Histopathology in patients with HCM is characterized by disarray of the overall architecture of the hypertrophied myocytes, which appear branched and may be intermingled with a variable amount of interstitial fibrosis^1^. These changes may be patched and must be distinguished from the non-specific physiological disarrangement of the junctional area of the septum and the apex. The myocardial cell diameter is another important indicator of hypertrophy. Under normal conditions it ranges from 5–12 µm in diameter. Anything up to 20 µm may be indicative of mild hypertrophy. In moderate hypertrophy cardiocyte diameter is up to 25 µm and moderate to severe hypertrophy is usually between 25-30 µm. For diameters greater than 30 µm severe hypertrophy must be suspected.

Hypertrophic cardiomyopathy almost always present with cell hypertrophy^2^. Hypertrophic myocardial cells show nuclear enlargement, bizarre nuclei and binucleation. Three patterns of fibrosis may be distinguished: interstitial or plexiform, replacement or scar fibrosis, and perivascular fibrosis. In interstitial diffuse fibrosis, bundles of collagen surround the cardiomyocytes individually. This is a non-specific pattern that may appear in other causes of heart failure.

Small arteries in the myocardium often show dysplastic, thickened walls, affecting mainly the medial and intima layers. As a consequence, an impairment of the vasodilatory capacity of the coronary arterioles occurs, and the vascular lumen appears abnormally reduced, leading to small vessel ischemia. Myocyte cell death and repair in the form of replacement fibrosis subsequently develop. Perivascular fibrosis spreads radially around capillary and small arteries. Replacement by adipose tissue is often seen in the terminal stage of fibrosis.

Ultra-structurally, HCM is characterized by an increase in the number of mitochondria, often without qualitative or quantitative signs of insufficiency. Enlargement of sarcoplasmic tubules, prominence of the Golgi apparatus and eventual accumulation of glycogen may be seen.

## Pathological differential diagnosis of HCM

The macroscopic finding of left ventricular hypertrophy (LVH) can be caused by a range of genetic or acquired conditions. Hypertrophic cardiomyopathy is most frequently a disease of the sarcomeric proteins. Mutations in several genes encoding thin and thick filament and the Z disk proteins of the sarcomeres (*MYH7, TPM1, TNNT2, MYBPC3, FHL1, CSRP3, FLNC, PLN)* have been described^3^. The histopathological appearance varies according to the genetic defect, with the most severe morphological abnormalities being present in pediatric and early onset cases^4^. Cellular hypertrophy of cardiomyocytes is commonly observed and fibrosis caused by fibroblast activation and collagen deposition may also be present.

CSRP3 (Muscle LIM Protein) MLP KO cardiomyocytes exhibit cytoarchitectural perturbations including disrupted myofibrillar assembly, abnormal alignment of Z-disks and marked fibrosis. In contrast, for some (but not all) HCM/RCM patients with Filamin C pathogenic variants, protein aggregation has been observed *in vivo* and *in vitro*^3^.

### Abnormal loading conditions

Hypertension and aortic stenosis are the most common acquired causes of LVH. On macroscopic examination, the thicknesses of the hypertrophied wall seen in hypertension rarely exceeds 1.5 cm. Valvular aortic stenosis results in concentric LVH, which is less usual in HCM where hypertrophy typically affects the interventricular septum in HCM. Approximately 30% of patients with HCM will also have right ventricular hypertrophy.

### Storage disorders

A rare cause of cardiac hypertrophy is Fabry disease (FD), an X-linked hereditary metabolic storage disorder that predominantly involves the heart^5^. The accumulation of glycosphingolipids, mainly globotriaosylceramide (Gb3), involves all cardiac tissues, including cardiomyocytes, vessels, cardiac valves, nerves and conduction system^6^. The ventricular hypertrophy in FD is usually concentric and frequently involves the right ventricle^7^.

Cardiac lysosomal storage of glycolypids gives rise to cardiomyocyte hypertrophy and a vacuolated appearance on light microscopy. Prominent sarcoplasmic vacuoles are present within the centers of myocytes and cause cellular enlargement. The vacuoles are clear, and occupy a variable proportion of the cardyomyocyte area, leading to confluent vacuoles in the most severe cases. They stain with Periodic Acid Schiff (PAS) and Sudan Black in frozen sections. Stromal diffuse fibrosis and fatty infiltration may also be present.

Under electron microscopy, enlarged lysosomes are typically located in the perinuclear area and contain electron-dense myelinoid inclusions with concentric lamellar configurations and a periodicity of 3 to 5 nm. These so-called ‘Zebra bodies’ are very suggestive of FD in patients without a history of chloroquine or amiodarone usage^8^. Lysosomal inclusions in FD may exhibit other morphologies, such as electron-dense homogeneous, radiated or mixed heterogeneous deposits. Immunohistochemistry with Gb3 is useful for confirming the nature of the stored material.

Other storage diseases that affect the heart, such as glycogen storage diseases (GSD), GM1 gangliosidosis and mucopolysaccharidoses (MPS) also result in cardiocyte hypertrophy. The most widely known glycogenoses involving the heart are type II (Pompe disease), type III (Cori disease) and type IV (Andersen disease).

The morphological consequences of the storage material are common to different entities, consisting of central vacuolar degeneration of myocytes, ranging from mild vacuolation to replacement of myofibrillar structures by large vacuoles, which appear pale pink with haematoxylin and eosin stain. Increased interstitial fibrosis may also appear^9^. Glycogen stains positive with periodic-acid Schiff (PAS), and is digested with diastase (PAS-D). The intralysosomal nature of the deposits in Pompe disease can be demonstrated by acid phosphatase staining on frozen tissue. Under electron microscopy, glycogen deposits are visualized as electrondense granules which appear membrane-bound within the cytoplasm of different cell types in Pompe disease, or free in the cytoplasm in other GSD.

In glycogenosis type IV, deficiency of glycogen branching enzyme results in the accumulation of abnormal glycogen, with fewer branching points, and amylopectin-like polyglucosans in different tissues^10^, that stains strongly positive on PAS, but is characteristically resistant to diastase, therefore excluding other types of glycogen storage disease. Electron microscopy demonstrates cytoplasmic inclusions composed of undulating, randomly oriented, and delicate fibrils. Infiltration of clear and granular histiocytes within endocardium, myocardium, cardiac valves, conduction system and vessel walls is a common finding in mucopolysaccharidosis (MPS) type I^11,12^.

Danon disease is an X-linked cardiomyopathy caused by mutations in the lysosome-associated membrane protein 2 (*LAMP2*) gene**.** Biventricular hypertrophy, extensive vacuolization of cardiocytes and fibrosis are common in Danon disease. It was originally considered to be due to a glycogen storage defect^13^, although genetic, histological and ultrastructural analyses have revealed that disruption of autophagy is the probable underlying mechanism of Danon disease^14^.

### Amyloidosis

Cardiac involvement is the leading cause of morbidity and mortality in several types of amyloidosis, especially in primary light chain (AL) amyloidosis and in both wild-type and hereditary transthyretin amyloidosis. The heart is also occasionally involved in serum amyloid A type (AA) acquired amyloidosis and other rare hereditary types^15^.

The two main types of interstitial involvement of the myocardium by amyloid deposits are pericellular and nodular, although a continuous spectrum exists between these. Vascular involvement with deposits in the arterial walls is less frequently seen. With Congo red staining, amyloid fibrils yield apple-green birefringence under cross-polarized light microscopy. This remains the gold standard for identifying amyloid deposits. On electron microscopy, amyloid appears as randomly arranged extracellular non-branching fibrils of 7–10 nm in diameter^16^.

Amyloid typing is crucial for an appropriate therapeutic approach. The immunohistochemically assessment of the amyloid type remains the essential diagnostic approach, although there are some problematic issues concerning IHC detection of amyloid antigens, particularly kappa and lambda light chains in AL amyloid. The best results with IHC examination are reached in AA amyloidosis and in most cases of TTR amyloidosis^17^.

### Mitochondrial disease

Mitochondrial disease is a heterogeneous group of multisystemic disease due to mutations in nuclear or mitochondrial DNA. In the last years more than 50 genes associated with mitochondrial cardiomyopathy have been described^18^. Cardiomyopathy is very frequent in mitochondrial disorders, with a minimum occurrence of 20–30% that varies with age, being more frequent in neonates and children. Cardiomyocytes appear hypertrophic, with perinuclear vacuolization and perimisal fibrosis, in the absence of inflammatory cell infiltrates.

Subsarcolemmal aggregates of mitochondria may be present, giving the classical appearance of ragged red fibers. These fibers appear red in the modified Gomori trichrome stain and blue in succinate dehydrogenase (SDH) and NADH tetrazolium reductase staining. Histochemistry for cytochrome c oxidase may be weak or absent. Electron microscopic examination is useful in demonstrating subarcolemmal increased numbers of mitochondria with irregular cristae, which can show semicircular arrangements or paracrystalline inclusions^19^. Mitochondria size variability and rarefaction of myofibrils may also be present^20^. Respiratory chain defects must be ruled out by measuring the enzyme activities in the endomyocardial biopsy.

## When to perform an endomyocardial biopsy in HCM

Endomyocardial biopsy (EMB) is the gold standard for the identification of cardiac allograft rejection, myocarditis and infiltrative and storage disease^21^, but non-invasive imaging, genetic testing and biochemical analysis are often sufficient to make specific diagnoses with resorting to an EMB. The interpretation of EMB specimens requires a multidisciplinary approach, knowledge of the patient’s clinical history and an appropriate understanding of cardiovascular pathophysiology.

In Fabry disease, an EMB may be necessary in female patients with equivocal genetic and biochemical findings^22^. EMB can provide evidence for a non-genetic aetiology of HCM, especially in cardiac phosphorylase kinase deficiency. EMB is also helpful in the evaluation of prognosis and follow up of response to treatment^23^.

## References

[ref-1] Shirani J, Pick R, Roberts WC, Maron BJ (2000). Morphology and significance of the left ventricular collagen network in young patients with hypertrophic cardiomyopathy and sudden cardiac death. J Am Coll Cardiol.

[ref-2] Ishibashi-Ueda H, Matsuyama TA, Ohta-Ogo K, Ikeda Y (2017). Significance and value of endomyocardial biopsy based on our own experience. Circ J.

[ref-3] Ehsan M, Jiang H, Thomson KL, Gehmlich K (2017). When signalling goes wrong: pathogenic variants in structural and signalling proteins causing cardiomyopathies. J Muscle Res Cell Motil.

[ref-4] Shirani J, Pick R, Roberts WC, Maron BJ (2000). Morphology and significance of the left ventricular collagen network in young patients with hypertrophic cardiomyopathy and sudden cardiac death. J Am Coll Cardiol.

[ref-5] Kubo T (2017). Fabry disease and its cardiac involvement. J Gen Fam Med.

[ref-6] Takenaka T, Teraguchi H, Yoshida A (2008). Terminal stage cardiac findings in patients with cardiac Fabry disease: an electrocardiographic, echocardiographic, and autopsy study. J Cardiol.

[ref-7] Yogasundaram H, Kim D, Oudit O (2017). Clinical features, diagnosis, and management of patients With Anderson-Fabry cardiomyopathy. Can J Cardiol.

[ref-8] Roos JM, Aubry MC, Edwards WD (2002). Chloroquine cardiotoxicity Clinicopathologic features in three patients and comparison with three patients with Fabry disease. Cardiovasc Pathol.

[ref-9] Takemura G, Kanamori H, Okada H (2017). Ultrastructural aspects of vacuolar degeneration of cardiomyocytes in human endomyocardial biopsies. Cardiovasc Pathol.

[ref-10] Andersen DH (1956). Familial cirrhosis of the liver with storage of abnormal glycogen. Laboratory Investigation.

[ref-11] Braunlin EA, Harmatz PR, Scarpa M (2011). Cardiac disease in patients with mucopolysaccharidosis: presentation, diagnosis and management. Journal of Inherited Metabolic Disease.

[ref-12] Hishitani T, Wakita S, Isoda T, Katori T, Ishizawa A, Okada R (2000). Sudden death in Hunter syndrome caused by complete atrioventricular block. J Pediatr.

[ref-13] Danon MJ, Oh SJ, DiMauro S, Manaligod JR, Eastwood A, Naidu S, Schliselfeld LH (1981). Lysosomal glycogen storage disease with normal acid maltase. Neurology.

[ref-14] Rowland TJ, Sweet ME, Mestroni L, Taylor MRG (2016). Danon disease – dysregulation of autophagy in a multisystem disorder with cardiomyopathy. Journal of Cell Science.

[ref-15] Guan J, Mishra S, Falk RH, Liao R (2012). Current perspectives on cardiac amyloidosis. American Journal of Physiology - Heart and Circulatory Physiology.

[ref-16] Flodrova P, Flodr P, Pika T (2018). Cardiac amyloidosis: from clinical suspicion to morphological diagnosis. Pathology.

[ref-17] Wechalekar A, Gillmore J, Hawkins P (2016). Systemic amyloidosis. Lancet.

[ref-18] Finsterer J, Kothari S (2014). Cardiac manifestations of primary mitochondrial disorders. Int J Cardiol.

[ref-19] Meyers DE, Basha HI, Koenig MK (2013). Cardiac manifestations of mitochondrial disorders. Tex Heart Inst J.

[ref-20] Kuno T, Imaeda S, Asakawa Y, Nakamura H, Takemura G, Asahara D, Kanamori A, Kabutoya T, Numasawa Y (2017). Mitochondrial cardiomyopathy presenting as dilated phase of hypertrophic cardiomyopathy diagnosed with histological and genetic analyses. Case Rep Cardiol.

[ref-21] Crossman DJ, Ruygrok P, Hou YF, Soeller C (2015). Next generation endomyocardicial biopsy: the potential of confocal and super-resolution microscopy. Heart Fail Rev.

[ref-22] Smid BE, van der Tol L, Cecchi F (2014). Uncertain diagnosis of Fabry disease: consensus recommendation on diagnosis in adults with left ventricular hypertrophy and genetic variants of unknown significance. Int J Cardiol.

[ref-23] Yuan SM (2018). Cardiomyopathy in the pediatric patients. Pediatr Neonatol.

